# Fracture Strength of Mesiobuccal Roots Following Canal Preparation with Hand and Rotary Instrumentation: An In Vitro Study

**Published:** 2011-08-15

**Authors:** Maryam Zare Jahromi, Parvin Mirzakouchaki, Elnaz Mousavi, Amir Arsalan Navabi

**Affiliations:** 1. Department of Endodontics, Dental School, Islamic Azad University of Medical Sciences, Khorasgan, Isfahan, Iran.; 2. Department of Operative Dentistry, Dental School, Islamic Azad University of Medical Sciences, Khorasgan, Isfahan, Iran.

**Keywords:** Dental Equipment, Endodontics, Root Canal Preparation, Root Fracture, Tooth Fracture

## Abstract

**INTRODUCTION:**

The aim of this in vitro study was to compare the effect of hand and rotary instruments on fracture strength of tooth roots.

**MATERIALS AND METHODS:**

Thirty two teeth were randomly divided into two experimental groups of 15 each and one negative control group with two samples. In group 1, step-back technique with conventional stainless steel K-files was used, and in group 2, instrumentation was performed using rotary Ni-Ti Hero642. Samples in control group did not receive cleaning or shaping after access cavity preparation. After obturating each canal, tip of the spreader was locked within canal. The required force for root fracture was measured using Instron testing machine. Recorded data was statistically analyzed using t-test.

**RESULTS:**

The mean and standard deviation force required for vertical root fracture were 50.33±19.1 and 63.1±25.46 N for hand and rotary groups respectively. However, no significant difference was found between experimental groups.

**CONCLUSION:**

The results indicate that the manual technique did not lower fracture strength of obturated roots in comparison with the rotary preparation technique.

## INTRODUCTION

Vertical root fracture (VRF) is a challenging complication which may occur in endodon-tically treated teeth[1][2][3]. Prevalence of VRF ranges from 2-5%[1][4]. Most VRFs occur in a buccolingual direction[5][6] and may involve root or root and crown. The prognosis of VRF is very poor and fractured teeth are often extracted or hemisected[7]. Dentine structure of endodontically treated teeth is not more brittle than that of vital teeth[7][8][9], although one study showed that root canal treatment causes moisture loss in tooth dentin[10]. Access cavity preparation has no significant effect on fracture strength of a tooth[11], even though Panitvisai and Messer found that cuspal deflection increases in larger access cavities[12]. Loads generated during lateral condensa-tion were significantly lower than the required values for fracturing a tooth. Weakening effect of excessively large canal preparations may compromise the fracture strength of the roots[1][2][3][7][8][9].

Rotary nickel-titanium (Ni-Ti) instruments show less apical canal transportation and perforations during canal preparation. Furthermore, canals have a rounded or oval shape and remain more centered[13][14][15][16][17]. In order to gain these benefits, advanced instrument designs with non-cutting tips, radial lands, different cross-sections, and superior resistance to torsional fracture and varying tapers have been developed[18]. Most rotary Ni-Ti instruments have a taper larger than the ISO standard 0.02 taper design, ranging from 0.04 to 0.12[19]. Internal shape of root canals which are prepared with hand files are more irregular than those with Ni-Ti[14][17]. The aim of this in vitro study was to determine weakening effects of rotary canal preparation compared to manual techniques.

## MATERIALS AND METHODS

Human extracted upper first molars were stored in 5.25% NaOCl solution (Daropakhsh, Karaj, Iran) for 20 minutes. The ligaments were removed by ultrasonic scaler and examined for immature root apices, cracks on the root surface, canals, gross caries involving the root, and for exceptionally short, thin, or curved roots. Teeth with these characteristics were discarded. Thirty-two teeth with moderate curvature were selected. The curve of mesiobuccal roots was ≈25 degrees (according to Schneider 1971). The teeth were randomly divided into two groups of 15 samples each, and also one group of duplicate negative control were taken. Root canal preparation was performed as follows: Group 1 used step-back technique with conventional stainless steel K-files (Mani Inc., Takanezawa, Japan); Group 2 used rotary Ni-Ti Hero642 instruments (Micro-Mega, Besancon, France). The two remaining teeth were considered as the negative control in order to evaluate the accuracy of the method. In the negative control group, after providing the access cavity, no cleaning or shaping was performed. Access cavity was prepared and the working length of the mesiobuccal canal was determined using K-file size 10 up to the apex minuses 0.5mm. The first 15 samples were prepared manually with K-files in step-back technique up to master apical file size 25. In all processes RC Prep (Premier, USA) was used as a lubricant. Recapitulation with the master apical file at the working length was carried out after each step-back size file. The 15 samples of the second group were cleaned and shaped with rotary instruments using Hero642 system files according to instructions. In rotary group apical root preparation was performed up to size 25 as master apical file.

In all samples of the second group, rotary instruments were used in the crown-down technique and the rotating velocity was 500 rpm. As recommended by the manufacturer, each rotary instrument was used 10 times. In all steps, RC Prep was used as a lubricant. After changing each file, decontamination with 1% NaOCl was performed.

In the next step, canals were filled by lateral condensation using gutta-percha (Ariadent, Tehran, Iran) and AH26 sealer (Dentsply, Switzerland). Finally, radiographs were taken for observing the quality of filling. During all processes, the teeth were maintained in normal saline (Daropakhsh, Karaj, Iran) in incubator with same condition (37ºC and 100% humidity, which in turn facilitates setting. Approximately 2mm below the cemento-enamel junction, the teeth were cut and divided into crown and root using high speed diamond-fissure burs (Tizkavan, Tehran, Iran). Gutta-percha (GP) in canals orifices was removed to the depth of 2mm with Gates Glidden drills number 1 and 2; so that the spreader (Mani Inc., Takanezawa, Japan) would stand in vertical direction inside the orifice. This allowed the spreader tip to apply a vertical force down the root canal. The roots were mounted in putty (Zhermack, Rovigo, Italy) vertically. This facilitates the spreader to be situated vertically. The putty was allowed to set for at least 30 minutes before teeth were tested. The putty casts were kept in damp towels. In the final step, the tip of the spreader size 35 was fixed in the canal and a force applied to the root by Instron testing machine (Instron Worldwide Headquarters, Norwood, MA, USA), until it began to decrease on the machine’s monitor. The maximum force displayed on the monitor before it began to decrease was recorded, which indicates the necessary force for fractures that are even invisible with the naked eye. In negative control group, spreader size 30 was fixed vertically and examined under the force of the Instron machine. Finally, the data was statistically analyzed by t-test.

## RESULTS

The analysis showed that the highest resistance to fracture was in the negative control group. Also, the mean root resistance to fracture was 50.33±19.1 and 63.1±25.46 N in the manual and rotary groups (Table 1). Data were analyzed using the SPSS software (Chicago, IL, USA). The final recorded data were analyzed using paired t-test which show no statistical difference between manual and rotary groups (P>0.05). Statistical analysis was performed at the 95% level of confidence. Although rotary group included a slightly higher mean value compared to group 1, the difference was not shown to be statistically significant.

**Table 1:** Fracture strength (Newton) of mesiobuccal root in studied groups

**Group**	**N**	**Min.**	**Max.**	**Mean±SD**
**Manual**	15	31.5	108.5	50.33±19.01
**Rotary**	15	35	113.5	63.1±25.46
**Control**	2	114.5	121.5	118±4.94

## DISCUSSION

Fracture resistance of teeth after endodontic treatment is an important consideration in dentistry. The main causes of vertical fractures in teeth with root treatment are attributed to methods of cleaning and shaping and canal obturation. Generation of force within the canal space by means of a spreader inserted into the canal is an accepted way for investigating fracture resistance[6][7][20][21][22]. The spreader may bind directly against the canal wall or by means of gutta-percha. The load required to fracture the root is an indicator for fracture resistance of roots.

The slightly higher resistance in the rotating group could be the result of smooth walls created by the rotating system. On the other hand, in the rotating systems, canal centra-lization is more favorably maintained. Using rotating method, the canal resistance to fracture may be slightly increased. However, Wilcox et al. study concluded that removing more root dentin led to more root fracture[22]. This may be a result of the effect of smoother and rounder canal shapes prepared by rotary Ni-Ti leading to reduced canal irregularities, which increase fracture susceptibility.

Lertchirakarn et al.[7] found the mean fracture load for mesial roots of mandibular molars to be 8.1 kg; the minimum load determined by Lindauer et al.[20] for a mandibular molar was 7 kg. The results of our study, 50.33±19.1 N (5.14±1.02 kg) and 63.1±25.46 N (6.44±2.6 kg) for hand and rotary preparations, respectively, had close similarities to the findings of Lertchirakarn et al. and Lindauer et al.’s studies[7][20]. However, our obtained values were considerably lower than the fracture loads in the study carried out by others. (10.2 kg for hand and 15.7 kg and 13.2 kg for LS and GT files). Pitts et al.[6] found the mean force required to fracture maxillary central incisors was 15.2 kg. Holcomb et al.[21] measured the fracture strength of mandibular incisors and found the minimum load required to fracture a mandibular incisor to be 1.5 kg.

We can suggest that teeth at risk of vertical root fracture might benefit from rotating systems for root canal preparations. Further studies on the effects of other rotating systems on fracture resistance of the teeth are also suggested.

## CONCLUSION

Manual canal preparation did not weaken the root structure more than rotary canal preparation. However, more studies with other rotary files and larger sample size are suggested.
